# Checkpoint-independent scaling of the *Saccharomyces cerevisiae* DNA replication program

**DOI:** 10.1186/s12915-014-0079-z

**Published:** 2014-10-07

**Authors:** Ariel Gispan, Miri Carmi, Naama Barkai

**Affiliations:** Department of Molecular Genetics, Weizmann Institute of Science, Rehovot, 76100 Israel

**Keywords:** mrc1, Checkpoint, Replication timing

## Abstract

**Background:**

In budding yeast, perturbations that prolong S phase lead to a proportionate delay in the activation times of most origins. The DNA replication checkpoint was implicated in this scaling phenotype, as an intact checkpoint was shown to be required for the delayed activation of late origins in response to hydroxyurea treatment. In support of that, scaling is lost in cells deleted of *mrc1*, a mediator of the replication checkpoint signal. Mrc1p, however, also plays a role in normal replication.

**Results:**

To examine whether the replication checkpoint is required for scaling the replication profile with S phase duration we measured the genome-wide replication profile of different MRC1 alleles that separate its checkpoint function from its role in normal replication, and further analyzed the replication profiles of S phase mutants that are checkpoint deficient. We found that the checkpoint is not required for scaling; rather the unique replication phenotype of *mrc1* deleted cells is attributed to the role of Mrc1 in normal replication. This is further supported by the replication profiles of *tof1Δ* which functions together with Mrc1p in normal replication, and by the distinct replication profiles of specific POL2 alleles which differ in their interaction with Mrc1p.

**Conclusions:**

We suggest that the slow fork progression in *mrc1* deleted cells reduces the likelihood of passive replication leading to the activation of origins that remain mostly dormant in wild-type cells.

**Electronic supplementary material:**

The online version of this article (doi:10.1186/s12915-014-0079-z) contains supplementary material, which is available to authorized users.

## Background

In eukaryotic cells, genome replication is initiated from multiple sites termed replication origins. The locations of those origins and their relative activation times during S phase are largely conserved between individual cells defining the DNA replication program [1–4]. In recent years, the DNA replication program was mapped in different organisms [5–9]. Early replication was found to correlate with low mutation rate [10], high gene expression, open chromatin and a reduced nucleosome abundance [2,8,11,12]. Yet little is known about the genetic determinants of origin replication times or firing efficiencies. Moreover, while the replication program is clearly reproducible at the level of cell populations, it is not clear whether individual cells activate the same origins in the same precise temporal order or whether origin firing is partly stochastic [13–17].

In the budding yeast, many perturbations which extend S phase have a small effect on the replication program since the activation times of most origins are delayed in proportion to S phase duration. This was first observed in cells subject to a non-lethal dosage of hydroxyurea (HU) [18] and later when analyzing the replication profiles of thirteen S phase mutants identified in an unbiased screen for cells with an extended S phase [19]. Those mutants were associated with aspects of DNA replication including the replication machinery, cell-cycle regulation and nucleotide metabolism. S phase was significantly extended in all mutants, but the genome-wide replication program was hardly altered: the identity of activated origins and their relative activation times (or efficiencies) remained essentially the same as those in wild-type. In most mutants, a minority of origins were lost [19]. Origin loss was reported previously in cells deleted of *clb5* [14,20].

This scaling of the replication program was not observed in just one perturbation, deletion of *mrc1*. Similarly to the other mutants, *mrc1* deletion extended S-phase. However, rather than losing origins, dormant origins were now activated so that a larger number of origins contributed to DNA replication. The resulting replication profile was sharper and was clearly distinct from all other profiles. A more recent study reported a similar sharpening of the replication profile in fission yeast deleted of the *mrc1* homologue [21].

Mrc1p plays a dual role in replication. First, Mrc1p functions in the replication checkpoint by mediating the DNA damage signal from the sensor Mec1p to the checkpoint kinase Rad53p. The possible involvement of the replication checkpoint in the scaling phenotype was suggested [18,19,22], since deletion of either *mec1* or *rad53* activated late origins that were inhibited by HU treatment [23–26]. This activation of late origins is reminiscent of the increased origin firing observed when *mrc1* was deleted. However, Mrc1p is found also at all unperturbed forks where it interacts with both the DNA helicase [27–31] and DNA polymerase epsilon [32], and promotes the coupling of polymerase progression to DNA synthesis [28]. Deletion of *mrc1* reduces fork progression rate [30,33,34]. Also, when replication is arrested by environmental stress such as HU, Mrc1p promotes the formation of a stable pausing complex [28].

In this study we asked which of the MRC1 functions is responsible for the unique replication profile of the *mrc1* deleted cells. To this end we first analyzed different *mrc1* alleles that separate its function in checkpoint signaling from its role in normal replication. Second, we examined S phase mutants that are also checkpoint-deficient and deleted of either the checkpoint kinase, *mec1* or the checkpoint sensor, *rad53*. Third, we analyzed cells deleted of *tof1* which cooperates with MRC1 during normal replication and cells expressing distinct *pol2* alleles differing by their interaction with Mrc1p. We find that scaling of the replication program does not require an intact checkpoint and provides evidence that the unique profile of *mrc1* deleted cells results from Mrc1p function in normal replication. We propose that sharpening of the replication profiles upon *mrc1* deletion is explained by the associated reduction in fork-velocity.

## Results

### The replication profile of cells deleted of MRC1

To enable a rigorous comparison of different replication profiles and, in particular, to define more rigorously the unique profile of *mrc1* deleted cells, we revisited the data we reported previously describing the replication profiles of mutants with an extended S phase [19]. These replication profiles were generated by microarray-based measurements of the DNA content of fluorescence-activated cell sorting (FACS)-sorted S phase cells (FACS profile in Figure S1, Additional file 1). To obtain higher resolution data and verify our previous results we repeated those experiments using high-throughput sequencing. The temporal replication profile is obtained by plotting the (normalized and smoothed) read-count as a function of the chromosomal coordinate. This profile describes the abundance of each genomic region in the S-phase population and, hence, correlates with its replication time or efficiency; loci that replicate early in S phase are present at high abundance in this asynchronous population, whereas the abundance of loci that replicate late is low. Replication origins are, therefore, detected as peaks in this profile, and their relative height indicates their typical firing time or efficiency.

**Figure 1:**
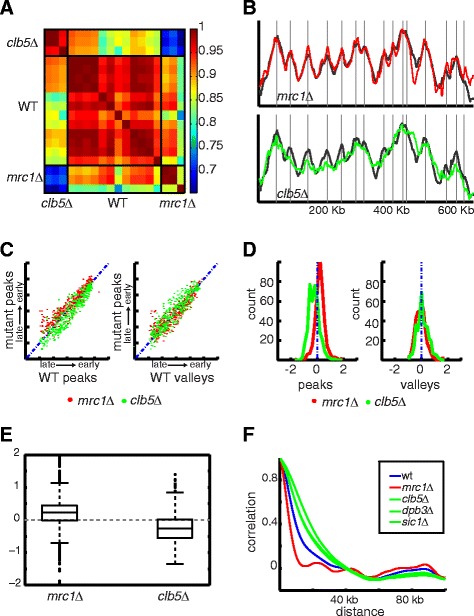
**Deletion of MRC1 sharpens the replication profile. A)**. Correlation matrix of the replication profiles*:* shown are the Pearson's correlation coefficients measuring the similarity between the replication profiles of wildtype cells (seven biological repeats), *mrc1* deleted cells (three repeats) and mutants previously associated with the scaling phenotype (*clb5Δ*, dpb3*Δ*, *dpb4Δ*, *dia2Δ*, *met7Δ*, *and sic1Δ*). Note the low correlation of the *mrc1* profile with the profiles of the scaling mutants. **B)**. Replication profiles at chromosome XI. DNA was extracted from FACS-sorted asynchronous S phase population and quantified using high-throughput illumina sequencing. The average DNA content measured at each genomic position is plotted as a function of the chromosomal coordinates. Active origins appear as local maxima. Plotted are replication profiles of *mrc1Δ* (red) and *clb5Δ* (green), with wild-type in black. Gray vertical lines represent location of confirmed origins, as defined in OriDB. **C)**. Gene deletion alters the relative origin activation time: the relative activation time of each OriDB-defined origin (*f*_*strain (xi)*_ *,* peak height of the respective replication profile) was measured from the respective profile and is shown as a function of the relative activation time of the respective origin at wild type, *f*_*wt (xi)*_ (left subplot, red denotes *mrc1Δ*, green denotes *clb5Δ*). Left subplot shows the same for local minima. **D)**. Profile sharpness defined by height of local maxima and local minima: Shown are the histograms of the function (f_strain (xi)_ –f_wt (xi)_) displayed for each strain (red = *mrc1Δ*; green = *clb5Δ*) for local maxima (left) and local minima (right). The respective Boxplots are shown in **E)** and represent the percentiles 75%, 50% and 25%. **E)**. Boxplot of the data in **C)**. **F)**. Profile sharpness defined by decay of autocorrelation: Plot of the autocorrelation of wildtype, *mrc1Δ*, *clb5Δ*, *dpb3Δ* and *sic1Δ* in blue, red green, green and green, respectively. FACS, fluorescence-activated cell sorting.

We previously reported that the replication profile of *mrc1* deleted cells is unique and we now verified it again using the sequencing-based profiles (Figure 1A). Visual inspection suggested that the replication profile of *mrc1* deleted cells is significantly sharper than that of the wild-type profile (Figure 1B, Figure S2 in Additional file 1). As we described previously, this sharpness results from strong activation of origins that remain largely dormant in wild type cells. Many of the new peaks in the *mrc1* profile are listed as confirmed origins in OriDB [35], and careful visualization of the wild-type profile suggests that they are weakly activated also in wild-type. The sharpness of each replication profile can be quantified by the heights of the local maxima, and by the depths of the local minima (Figure 1C, D, E). It can also be measured by plotting the autocorrelation function describing the typical chromosomal distance over which DNA abundance correlates (Figure 1F). This sharpening of the profile was unique to *mrc1* deleted cells and was not observed in any of the other S-phase mutants we tested. In fact, the other mutants showed the opposite phenotype, being smoother than the wild-type profile by both our criteria: lower local maxima and a slower-decaying autocorrelation. (Figure 1C-F; data shown only for a representative *clb5* deletion).

Notably, known replication origins were best predicted by the *mrc1Δ* profile, increasing the number of correctly identified origins from 178 in wild type cells (178/194 hits/peaks; *P*-value of 10^−70^ ) to 213 in *mrc1* deleted cells (213/238, *P* =10^−78^). Predictions based on profiles of the other mutants tested were equivalent, or worse than the wild-type prediction. Together, these observations indicate that while most mutants display a smoother profile characterized by a lower number of active origins, deletion of *mrc1* sharpens the profile, with a larger number of origins significantly contributing to DNA replication.

### The replication phenotypes of different mrc1 alleles that differently influence DNA checkpoint activation

Mrc1 has a dual role in replication, acting as a mediator of the replication checkpoint response during replication stress and also assisting fork progression during normal replication. We examined four alleles that were reported to differentially affect the Mrc1 role in checkpoint activation: the *mrc1-aq* allele is mutated in all of its phosphorylation sites and is incapable of mediating the checkpoint signal [36]. Similarly, the *mrc1-N5* allele generated through a partial N-terminal deletion interacts synthetically with *rad9* deletion, indicating a role in the checkpoint response [37]. We further examined two *mrc1* alleles obtained by C-terminal deletions: *mrc1-C14* and *mrc1-C15.* Those mutations extend S-phase but are not synthetic lethal with *rad9Δ* or *rrm3Δ* and show normal HU resistance, suggesting an intact checkpoint. Therefore, those mutations impact on MRC1 function during normal replication [37].

**Figure 2:**
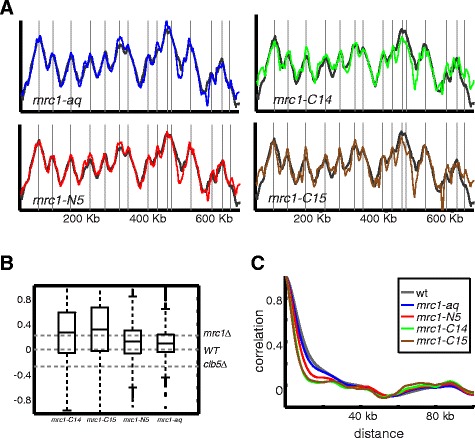
**Replication profiles of MRC1 alleles suggest that its unique phenotype reflects its function in normal replication. A)**. Replication profile of chromosome XI*.* Same as Figure 1A for the indicated strains (*mrc1-aq, mrc1-N5, mrc1-C14, mrc1-C15*). **B)**. Profile sharpness defined by height of local maxima and local minima: Same as Figure 1D for the indicated strains. **C)**. Profile sharpness defined by decay of autocorrelation: Same as Figure 1E for the indicated strains.

The replication profile of *mrc1-aq* was highly similar to the wild-type, while that of *mrc1-N5* was an intermediate between the wild-type and full *mrc1*deletion (Figure 2). Perhaps more informatively, both c-terminal mutants, which left the checkpoint intact, displayed a phenotype that was highly similar to that of *mrc1-*deletion and, in fact, was significantly sharper than the checkpoint deficient alleles (Figure 2)*.* These results suggest to us that the unique replication profile of *mrc1*deleted cells occurs even in the presence of intact checkpoint.

### Impaired checkpoint does not explain the mrc1Δ phenotype

To further examine the possible role of the DNA replication checkpoint in the scaling of the replication profile with S phase duration, we tested more directly the replication profiles of mutants deficient in replication checkpoint function. The checkpoint is impaired in strains deleted of the DNA damage sensor *mec1* or the checkpoint kinase *rad53* [38]. Deletion of *mec1* or *rad53* is lethal, but can be rescued by the deleting *sml1*, an inhibitor of the RNR enzyme regulating dNTP production [39].

**Figure 3:**
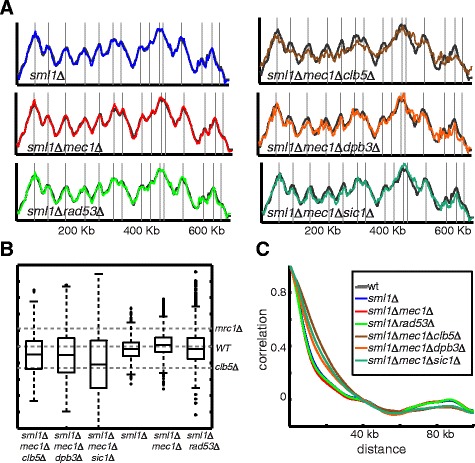
**Replication profiles of checkpoint mutants suggest that scaling does not require an intact checkpoint. A)**. Replication profile of chromosome XI. Same as Figure 1A for the indicated strains (*sml1Δ, sml1Δ mec1Δ, sml1Δ rad53Δ,sml1Δ mec1Δ clb5Δ, sml1Δ mec1Δ dpb3* and *sml1Δ mec1Δ sic1Δ*). **B)**. Profile sharpness defined by height of local maximas and local minimas: Same as Figure 1D for the indicated strains. **C)**. Profile sharpness defined by decay of autocorrelation: Same as Figure 1E for the indicated strains.

The replication profiles of the single or double deletion mutants (*sml1Δ*, *sml1Δ mec1Δ*, and *sml1Δ rad53Δ*) were practically indistinguishable from the wild-type profile by all our measures (Figure 3). Since those cells had a normal S phase, we additionally deleted genes that extended S phase (*clb5Δ, sic1Δ, dpb3Δ*) in the background of *mec1Δsml1Δ* deleted cells, generating three triple-mutant strains in which S phase is extended and the replication checkpoint is impaired. We examined the replication profile of the three mutants. The profiles were strongly correlated with those of the single deletion (*clb5, sic1, dpb3*) profiles, showing the same scaling-like phenotype. Correlation of those profiles to the profile of the *mrc1* deleted strain was low (Figures 3). Together, these results indicate that the checkpoint is not required for the scaling of the replication profile with S phase duration and that the replication phenotype of *mrc1* deleted cells is not connected to its role in mediating the checkpoint response.

### The mrc1-deletion phenotype is recapitulated by mutations that corroborate mrc1 function in normal replication

**Figure 4:**
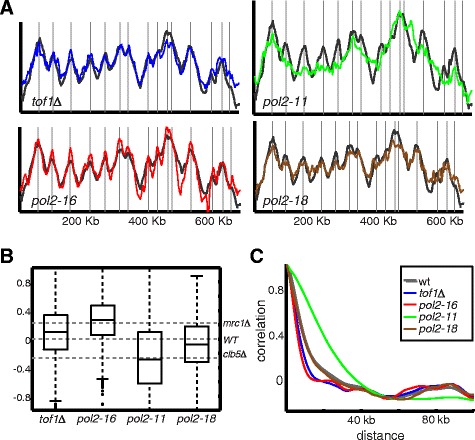
**Replication profiles of mutants impairing MRC1 function sharpen the replication profile. A)**. Replication profile of chromosome XI*.* Same as Figure 1A for the indicated strains.( *tof1Δ, pol2-11, pol2-16, pol2-18*). **B)**. Profile sharpness defined by height of local maximas and local minimas: Same as Figure 1D for the indicated strains. **C)**. Profile sharpness defined by decay of autocorrelation: Same as Figure 1E for the indicated strains.

Mrc1p is present in unperturbed replication forks where it interacts with the DNA polymerase. The Tof1p protein is required for Mrc1p function in normal replication but not for its checkpoint-associated role [28,40]. Examining the replication profiles of cells deleted of *tof1*, we find it to be sharper than the wild-type profile, being more similar to the profile of *mrc1* deleted cells than to the profile of the other mutants (Figure 4A-C).

Mrc1p interacts with DNA polymerase *ε* through both the C- and N- terminals of the catalytic POL2 subunit [32]. In the presence of HU the C-terminal interaction persists, indicating that it is independent of the checkpoint, whereas the N-terminal interaction is lost. It was suggested that MRC1p links the polymerase with the DNA helicase and in this way facilitates the progression of the replication fork under normal conditions and ensures the coordinated motion of the helicase and polymerase when nucleotide pulls are depleted under HU treatment [32]. We, therefore, asked whether mutations in the polymerase *ε* itself which impair its interaction with MRC1 will result in a replication profile similar to that of the *mrc1Δ* cells.

We considered three temperature-sensitive *pol2* alleles: *pol2-16*, *pol2*-*18* and *pol2-11*. All three mutants display an extended S-phase. In addition, all three are synthetic lethal with *mrc1*deletion [36]. *pol2-16*, which lacks the N-terminal (residues 176 to 1,134), is checkpoint proficient but displays a slow progression of the replication fork [41], while *pol2–11*, which lacks a fraction of its C-terminal, is checkpoint deficient [32,42]. Finally, *pol2–18*, which has a mutation in the N-terminal catalytic domain pro710Ser, is synthetic lethal with *rad9Δ,* indicating a role in the checkpoint response [32].

Notably, while S phase was equally extended in all three mutants (not shown), their temporal replication program was distinct. The profile of *pol2-16* allele was most similar to that of *mrc1Δ* deleted cells, consistent with its association with Mrc1p during normal replication. *pol2–11* on the other hand displayed the opposite phenotype characteristic of most non-mrc1 mutants and was more correlated with the profile of the *clb5Δ* cells, again consistent with our conclusion that the checkpoint is not required for the scaling phenotype. Finally, the *pol2–18* profile most closely resembled the wild-type profile, indicating the most efficient scaling of its temporal replication program with S phase duration (Figure 4A-C).

## Discussion

### Replication checkpoint does not inhibit origin firing in cycling cells

Our results demonstrate that deletion of *mrc1* sharpens the replication profile not because it impairs the function of the DNA replication checkpoint, but because it impairs the normal progression of the replication fork. This strongly argues that the replication checkpoint is not required for the scaling of the replication program with S phase duration observed in multiple S phase mutants.

Previous studies reported that deletion of *rad53* re-activates late origins which are inhibited by HU treatment [23,24]. This phenotype is similar to the activation of dormant origins observed in *mrc1*-deleted cells. We, therefore, began our study by hypothesizing that the *mrc1* deletion phenotype reflects lack of scaling due to an impaired checkpoint. Our results, however, largely refute this hypothesis. In fact, we found that perturbing the checkpoint in cycling cells does not increase the number of active origins and does not alter scaling. For example, deleting the checkpoint sensor *mec1* in multiple mutant backgrounds that extended S phase duration maintained the approximate scaling of the replication profile with S phase duration, and decreased rather than increased the number of active origins contributing to DNA replication.

Similarly, in our data, deletion of *rad53* did not significantly alter the replication program. Rather, cells maintained a replication profile that was largely similar to that of wild-type cells. It should be noted that our assay differed from the one used previously to characterize the Rad53p role that we considered unsynchronized S-phase population. In contrast, previous studies synchronized the cells and analyzed them at specific time points following release from cell-cycle arrest, not accounting for possible differences in the progression rates through S phase [23].

### Mrc1 phenotype reflects its function in normal replication

We provided evidence that the *mrc1Δ* phenotype is due to its function in normal replication. Mrc1p interacts with Pol2p, and its deletion is known to reduce fork velocity. Similarly, fork velocity is reduced also in the *pol2-16* allele, and we, indeed, found that the temporal replication program in cells carrying this allele is highly similar to that of *mrc1* deleted cells. A similar phenotype was found also when deleting *tof1* which assists MRC1 function in normal replication.

Reduced fork velocity likely increases the number of origins that are activated, as it reduces the likelihood of passive replication by a fork emanating from a nearby origin. Therefore, more origins will be activated, as is indeed observed in the *mrc1* deleted cells. In this scenario, *mrc1* deletion does not affect the identities of the origins or their initiation rate, but simply reduces the length of the typical region replicated between two initiation events. S phase becomes longer simply because it now takes longer to complete replication, although more origins are involved in the replication process.

## Conclusions

### Interpreting replication profiles of S phase mutants

Mutations that alter S phase are instrumental in revealing the molecular mechanism defining the replication program. The finding that most S phase perturbations delay origin activation in proportion to S phase duration suggests that this program is regulated by global factors that equally affect most origins. Despite this overall scaling, however, there are clear differences between individual mutants reflected in the apparent decreased efficiency of late, or less efficient origins [19]. This decreased efficiency is more apparent in some strains (for example, *clb5* delete) than in other strains. Furthermore, the relation between S phase extension or distribution and the associated replication profile is not clear; for example both *mrc1* and *clb5* deleted cells extended S phase to the same extent, yet show opposite replication profile phenotypes. Similarly, the S phase distribution of the different mutants we analyzed, as captured by the associated flow-cytometer profiles, varies. Those differences are difficult to interpret in the absence of a mechanistic model. Several models were suggested to explain the mechanistic basis of the DNA temporal replication program, invoking either a temporal regulation of origin firing, or a stochastic firing with some origin-specific probabilities [3,43,44]. While those models were evaluated with respect to their ability to predict the wild-type replication profile, they should be instrumental in defining the expected differences in the replication profiles upon S phase perturbations, and thereby interpreting mutant effects in a more rigorous manner.

## Methods

Strains used in this study are listed in Additional file 2: Table S1.

### Strain construction

Double deletions strains: BY4741 *sml1Δ::*kanMX, *rad53Δ:: hygromycinB,* BY4741 *sml1Δ::*kanMX, *mec1Δ:: hygromycinB,* were generated by amplifying the kanMX gene of the plasmid pBS7 (Yeast Resource Center) replacing SML1 orf by transformation. Second transformation was done by amplifying the Hygromycin-B gene of the plasmid pBS35 (Yeast Resource Center), replacing RAD53 orf or MEC1 orf by transformation.

We could not obtain triple haploids with *sml1Δrad53Δ* and only triple haploids of *sml1Δmec1Δ* with: *clb5Δ, sic1Δ* and *dpb3Δ* were obtained.

All deletion mutants were verified to be correct clones by colony PCR and sequencing.

### Growth conditions

Strains used in this study are listed in Additional file 2: Table S1. The medium used was yeast extract peptone dextrose (YPD) supplemented with Geneticin (200 μg/mL) (GIBCO, Rhenium Modi’in, Israel) or nourseothricin (Nat) (100 μg/mL) (Werner Bioagents, Jena, Germany) or hygromycin-B (300 μg/mL) when required. In all replication profiling experiments the cells were grown in YPD overnight, diluted to OD_600_ 0.1 in YPD, grown at 30 °C and harvested at OD_600_ 0.5.

### FACS staining

A total of 50 ml logarithmic cultures were fixated with 70% ethanol. Fixated cells were washed twice with 50 mM Tris–HCl (pH 8), treated with 6 ml of 1 mg/mL RNase A (Sigma, Rehovot, Israel) for 40 minutes at 37°C, washed twice with Tris–HCl, treated with 1 ml of 20 mg/mL proteinase K (Sigma) for one hour at 37°C, washed once with Tris–HCl and incubated for one hour at room temperature with 4 ml 1:1000 SYBR Green I (Invitrogen, Rhenium Modi’in, Israel).

### FACS sorting

Sorting was performed with the Beckton-Dickinson FACSAria sorter at minimal flow rate and sorting speed of approximately 20,000 to 30,000 cells/sec. Five million cells were sorted from each of G_1_/G_2_ and S phases.

### Library preparation and sequencing

DNA was digested with DpnII and multiplexed as previously described [45].

The resulting tagged library was sent to sequencing using an Illumina HiSeq 2500.

### Data processing

The sequencing reads were aligned to the cerevisiae genome using bowtie software and then grouped according to the expected DNA fragments resulting from DpnII cleavage (GATC). We have removed the following data points:

- Data points representing fragments shorter than 150 bp.
- Data points representing fragments that failed to align in the W303 strains (specified in the supplementary matlab code).
- Data points with zero reads in more than 300 different experiments.

Experiments with fewer than 100,000 reads were removed. Chromosomal duplications and deletions were normalized. Each experiment was normalized to mean 0 and std of 1, and the smoothed length bias (number of reads as a function of fragment length) was removed. The whole data matrix was divided in the mean G1/G2 signal. The data were decomposed using Singular Value Decomposition (SVD), and composed again only from the leading 50 vectors.

The signal was smoothed using Savitzky–Golay filter.

All processing process is available in the matlab code in Additional file 3.

### Prediction accuracy *P*-value calculation

We refer to the peaks (local maxima) of a profile as a prediction of origins. This was compared to a known origins reference list. For each profile, there are N predicted origins (peaks) that we ask how many of them are real origins. A predicted origin was defined as ‘real’ if it was located within 5 Kb to some known origin (confirmed and likely origins in OriDB [35]). We denote p the fraction of the genome that is covered by these 5-Kb windows (*P* =0.38 in our case). Under the assumption that predicted origins are randomly distributed (H_*0*_), the probability for a random location to be considered as an origin is *P*. Letting N be the total number of predicted origins and K the number of those defined as ‘real’, we calculate the *P* value, which is the probability to randomly choose N genomic locations that K or more of them are considered as real origins, using the binomial distribution:

$$ P\;\left(X\ge K\;\left|{H}_0\right.\right)={\displaystyle \sum_{i=K}^N\left(\begin{array}{c}\hfill N\hfill \\ {}\hfill i\hfill \end{array}\right)}{p}^i\;{\left(i-p\right)}^{N-i} $$

## Availability of supporting data

The microarray data have been submitted to the Gene Expression Omnibus (GEO) under accession number GSE32002.

The sequencing data have been submitted to the Sequence Read Archive (SRA) under accession number SRP049026.

## Additional files

Additional file 1:
**FACS profile and replication timing of all chromosomes for all the strains used in the paper.**
Additional file 2:
**Strains used in this study.**
Additional file 3:
**Matlab script used for data processing.**

## Abbreviations

FACS: fluorescence-activated cell sorting
HU: hydroxyurea
YPD: yeast extract peptone dextrose
