# Supplementary material for: Checkpoint-independent scaling of the Saccharomyces cerevisiae DNA replication program
Source: BMC Biol. 2014 Oct 7;12:79. doi: 10.1186/s12915-014-0079-z (PMC4218987; doi:10.1186/s12915-014-0079-z)
Supplement: Additional file 1: — FACS profile and replication timing of all chromosomes for all the strains used in the paper. [file 12915_2014_79_MOESM1_ESM.docx]

**supplementary information**

**Part A: mutant phenotypes**

- *mrc1aq:* mutation of the 17 TQ/SQ sites to AQ, preventing phosphorylation. Reduced viability in the presence of HU, or UV radiation. Failure to arrest cell cycle and to activate Rad53 . Normal progression during unpertubed S phase. There is no effect on localization on the replication fork (Osborn & Elledge, 2003)
- *mrc1n5:* Deletion of residues 1-359 of the MRC1 protein. Compromised growth with synthetic lethality with rad9Δ. Slight 5-min delay through S phase compared with WT.(Naylor, Li, Osborn, & Elledge, 2009)
- *mrc1c14:* Deletion of residues 844-1096 of the MRC1 protein. Normal growth on HU plates compared with MRC1. Rad53 activation is slightly delayed and less intense compared with WT. Delayed S-phase progression similar to that of mrc1Δ mutants. Rad53 activation during normal S phase.(Naylor et al., 2009)
- *mrc1c15:* Deletion of residues 904-1096 of the MRC1 protein. Normal growth on HU plates compared with MRC1. Delayed S-phase progression similar to that of mrc1Δ mutants. Rad53 activation during normal S phase.(Naylor et al., 2009)
- *pol2-11:* C-terminal mutation of POL2. Defective in DNA replication and in the cellular response to DNA damage during S phase.(Dua, Levy, & Campbell, 1998; Lou et al., 2008)
- *pol2-16:* The pol2-16 mutation has a deletion in the catalytic domain of DNA polymerase ε that eliminates its polymerase and exonuclease activities. Slow growth, and slow replication fork movement. (Lou et al., 2008; Ohya et al., 2002)
- *pol2-18:* temperature-sensitive mutation in the N-terminal catalytic domain of POL2. High viability in HU (Dua et al., 1998; Lou et al., 2008)

**Figure S1**: *Flow cytometry data – DNA content distribution for all mutants*

**
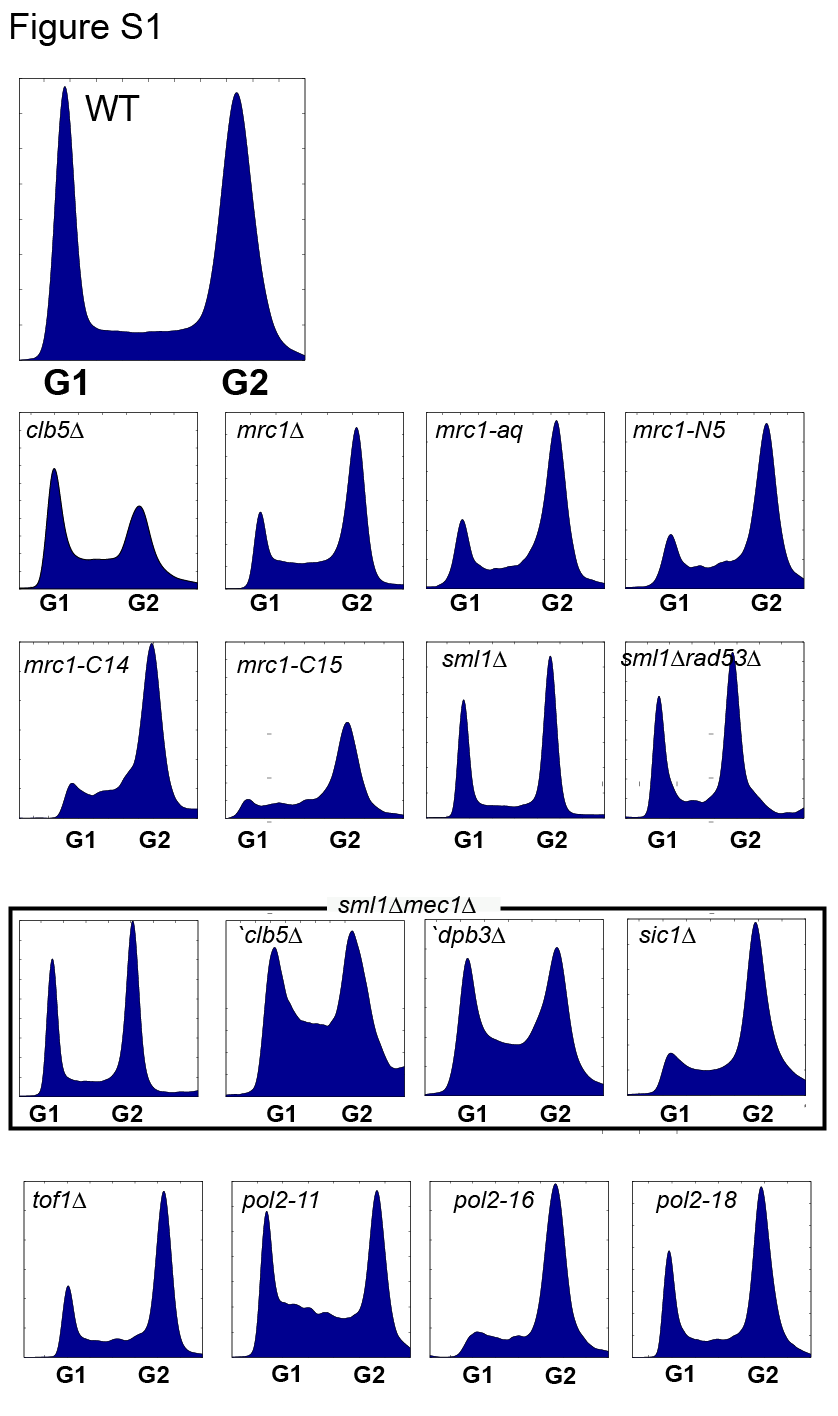
**

**Figure S2**: *Replication profiles presented in the paper - for all chromosomes*


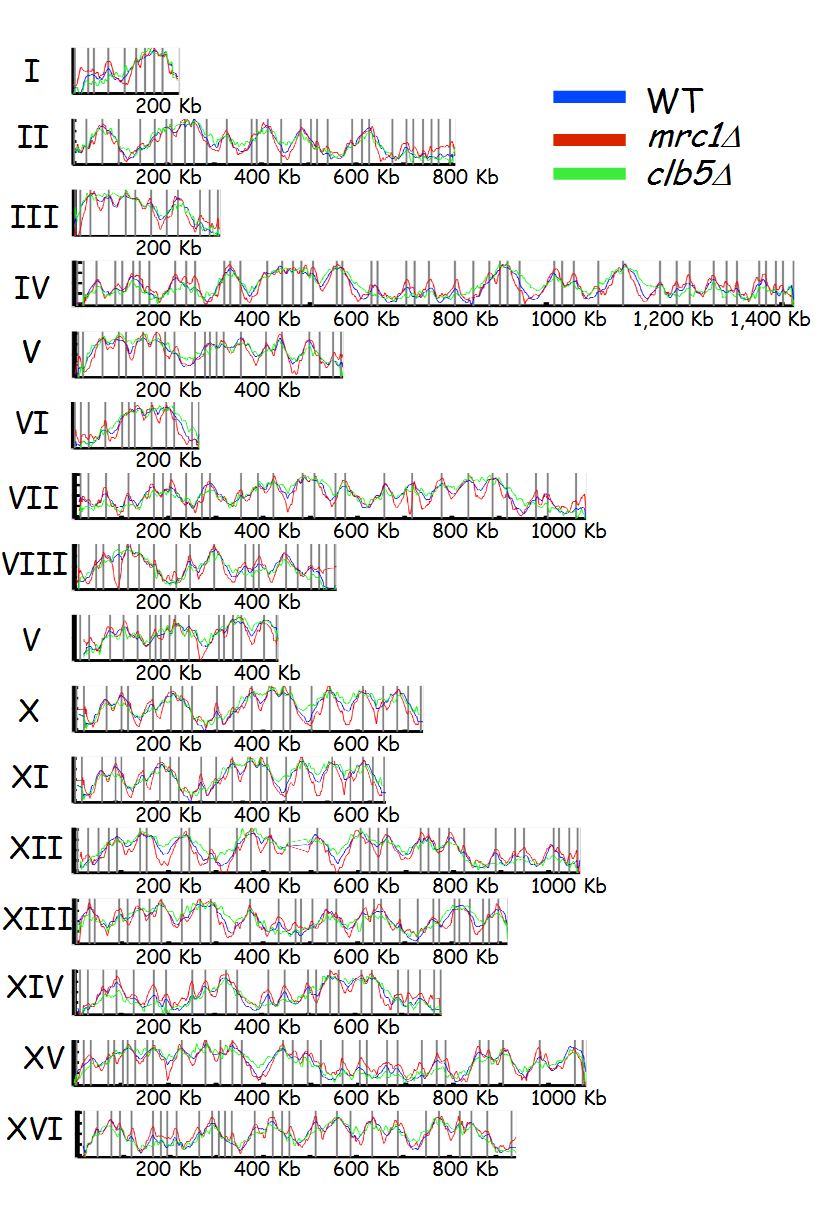


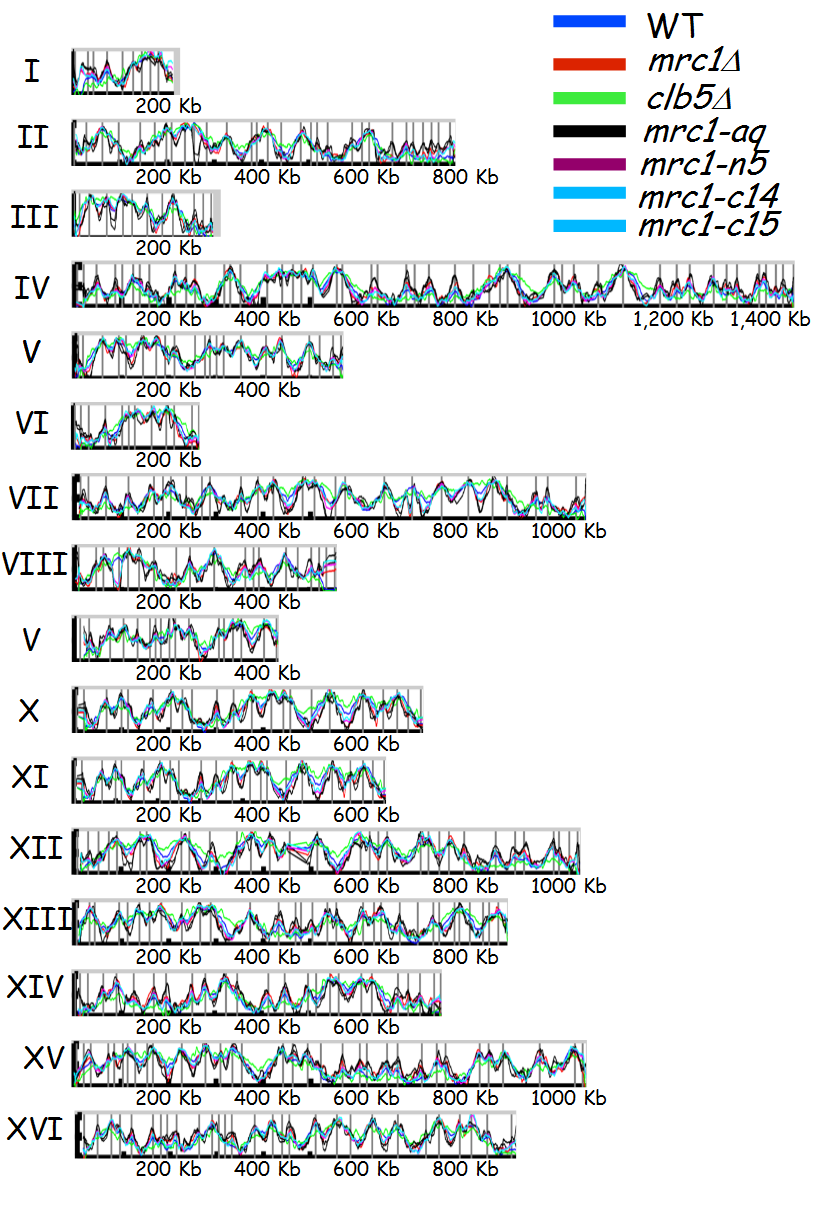


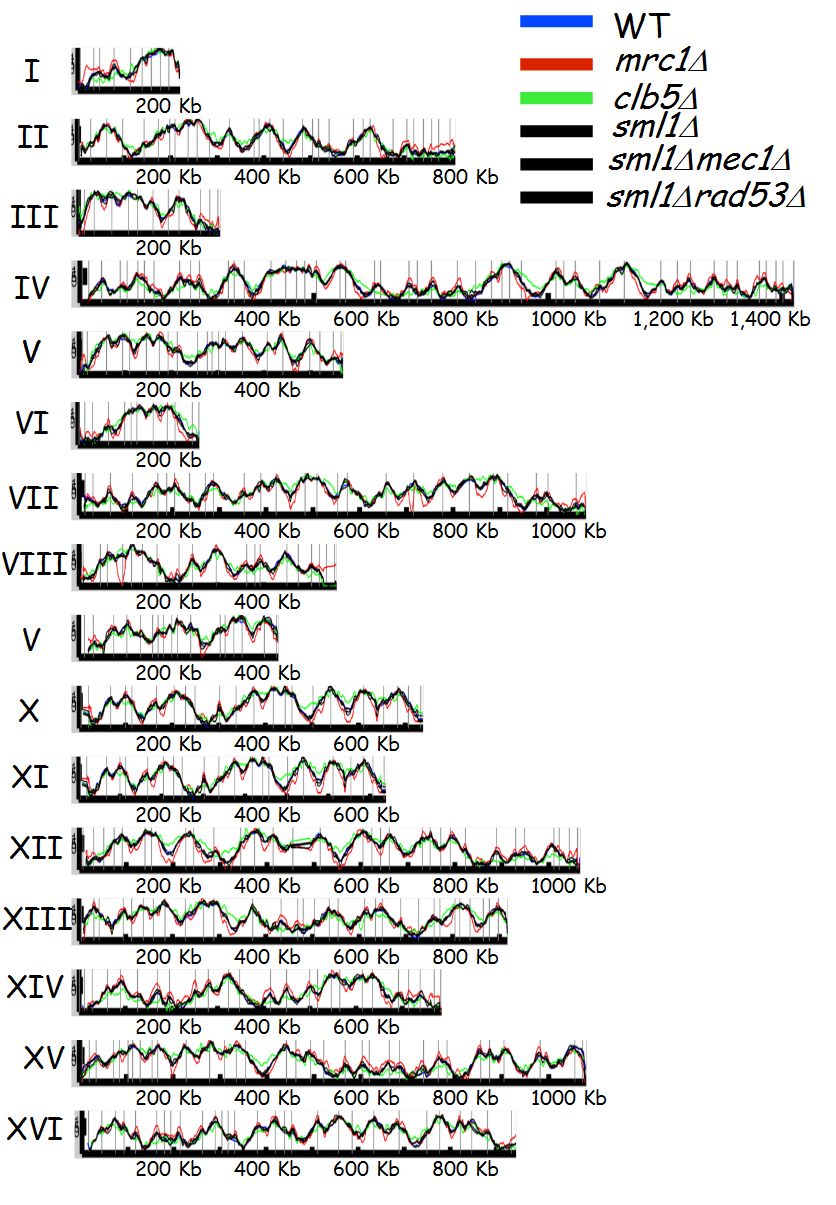


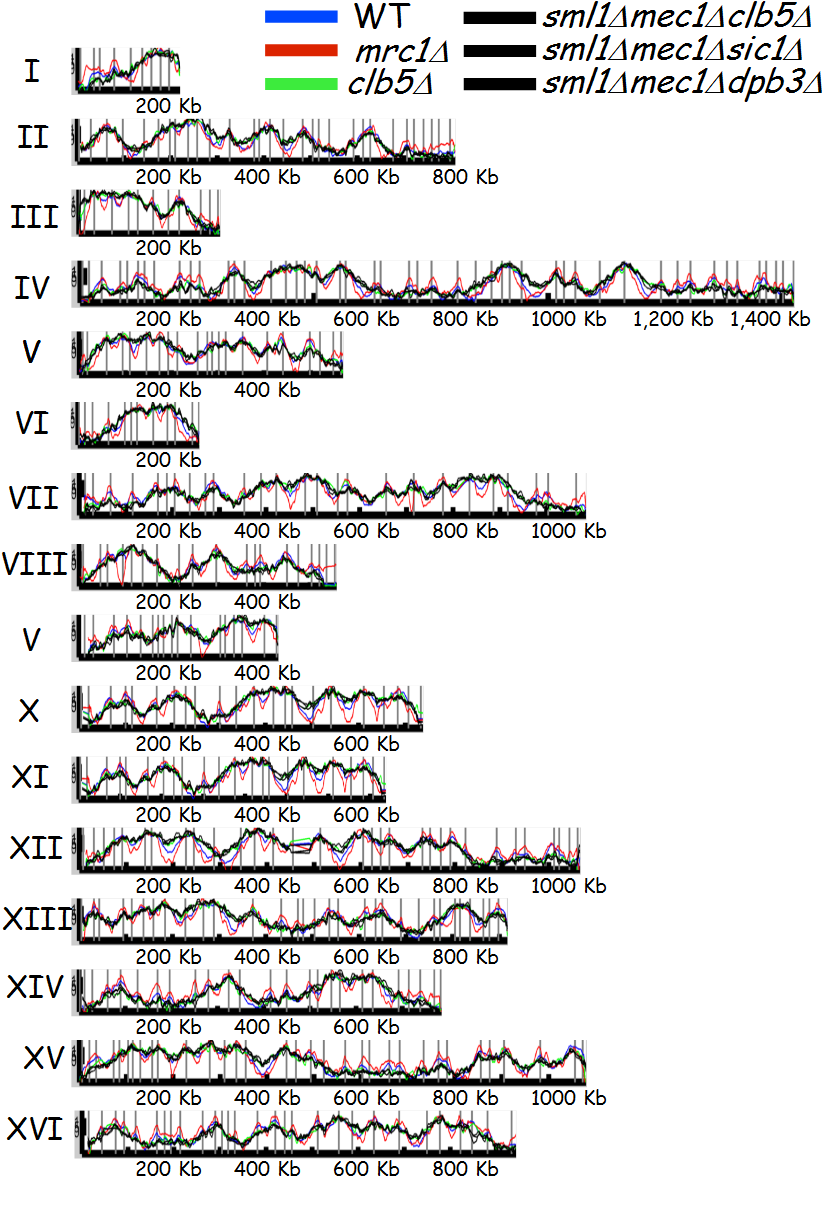


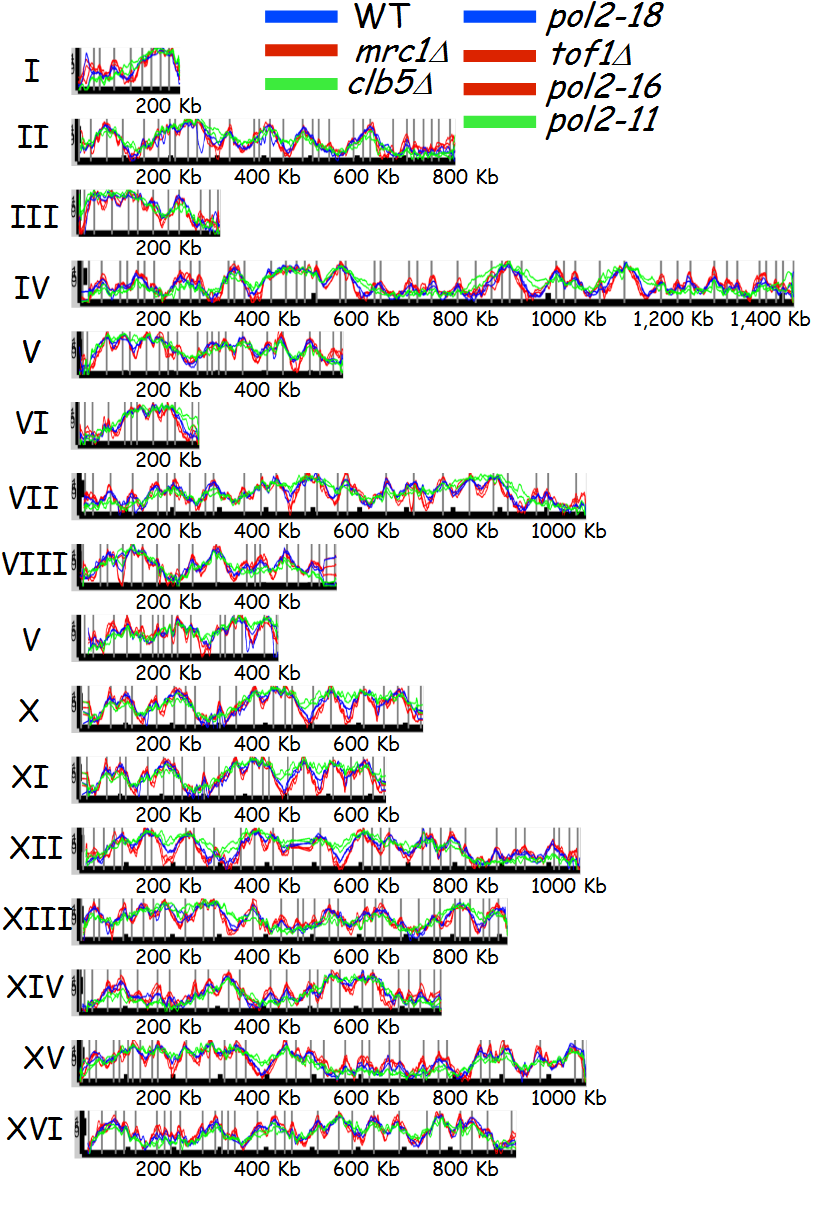


**References**

Dua, R., Levy, D. L., & Campbell, J. L. (1998). Role of the putative zinc finger domain of Saccharomyces cerevisiae DNA polymerase epsilon in DNA replication and the S/M checkpoint pathway. *J Biol Chem*, *273*(45), 30046–30055. Retrieved from http://www.ncbi.nlm.nih.gov/pubmed/9792727

Lou, H., Komata, M., Katou, Y., Guan, Z., Reis, C. C., Budd, M., … Campbell, J. L. (2008). Mrc1 and DNA polymerase epsilon function together in linking DNA replication and the S phase checkpoint. *Molecular Cell*, *32*(1), 106–117. doi:S1097-2765(08)00608-4 [pii] 10.1016/j.molcel.2008.08.020

Naylor, M. L., Li, J. M., Osborn, A. J., & Elledge, S. J. (2009). Mrc1 phosphorylation in response to DNA replication stress is required for Mec1 accumulation at the stalled fork. *Proc Natl Acad Sci U S A*, *106*(31), 12765–12770. doi:0904623106 [pii] 10.1073/pnas.0904623106

Ohya, T., Kawasaki, Y., Hiraga, S., Kanbara, S., Nakajo, K., Nakashima, N., … Sugino, A. (2002). The DNA polymerase domain of pol(epsilon) is required for rapid, efficient, and highly accurate chromosomal DNA replication, telomere length maintenance, and normal cell senescence in Saccharomyces cerevisiae. *J Biol Chem*, *277*(31), 28099–28108. doi:10.1074/jbc.M111573200 M111573200 [pii]

Osborn, A. J., & Elledge, S. J. (2003). Mrc1 is a replication fork component whose phosphorylation in response to DNA replication stress activates Rad53. *Genes Dev*, *17*(14), 1755–1767. doi:10.1101/gad.1098303 17/14/1755 [pii]
