# Supplementary material for: Checkpoint-independent scaling of the Saccharomyces cerevisiae DNA replication program
Source: BMC Biol. 2014 Oct 7;12:79. doi: 10.1186/s12915-014-0079-z (PMC4218987; doi:10.1186/s12915-014-0079-z)
Supplement: Additional file 2: — Strains used in this study. [file 12915_2014_79_MOESM2_ESM.docx]

**Table S1**

| ***strain*** | ***Repeats*** | ***Genotype*** | ***Source*** |
| --- | --- | --- | --- |
| *BY4741* | 12 | *MAT***a** *his3-*_*1 leu2-*_*0 met15-*_*0 ura3-*_*0* | This study |
| *mrc1Δ* | 1 | *BY4741 mrc1Δ::NAT* | This study |
| *mrc1Δ* | 2 | MATa trp1-1 ura3-1 his3-11,15 leu2-3,112 ade2-1 can1-100 mrc1::his5+ | [1] |
| *clb5Δ* | 3 | BY4741 clb5Δ::kanMX | [2] |
| *mrc1-aq* | 3 | *MATa trp1-1 ura3-1 his3-11,15 leu2-3,112 ade2-1 can1-100 HIS::mrc1AQ-MYC13* | [3] |
| mrc1-C14 (Y2544) | 3 | *MATa trp1-1 ura3-1 his3-11,15 leu2-3,112 ade2-1 can1-100 KANMX::mrc1-C14-MYC13* | [3] |
| mrc1-C15 (Y2545) | 2 | *MATa trp1-1 ura3-1 his3-11,15 leu2-3,112 ade2-1 can1-100 KANMX::mrc1-C15-MYC13* | [3] |
| mrc1-N5 (Y2553) | 3 | *MATa trp1-1 ura3-1 his3-11,15 leu2-3,112 ade2-1 can1-100 HIS::mrc1-N5-MYC5* | [3] |
| *sml1Δ* | 3 | BY4741 sml1Δ::kanMX | [2] |
| *mec1Δ, sml1Δ* | 3 | BY4741 sml1Δ::kanMX, *mec1Δ:: hygromycinB* | This study |
| *mec1Δ, rad53Δ* | 3 | BY4741 sml1Δ::kanMX, *rad53Δ:: hygromycinB* | This study |
| *mec1Δ, sml1Δ, clb5Δ* | 2 | *sml1Δ::kanMX*, *mec1Δ:: hygromycinB, clb5Δ::NAT* | This study |
| *mec1Δ, sml1Δ, sic1Δ* | 1 | *sml1Δ::kanMX*, *mec1Δ:: hygromycinB, sic1Δ::NAT* | This study |
| mec1Δ, sml1Δ, dpb3Δ | 1 | *sml1Δ::kanMX*, *mec1Δ:: hygromycinB, dpb3Δ::NAT* | This study |
| *tof1Δ* |  | BY4741 tof1Δ::kanMX | [2] |
| *pol2-11* | 4 | [*MATa*](http://db.yeastgenome.org/cgi-bin/locus.pl?locus=MATA) [*ade2*](http://db.yeastgenome.org/cgi-bin/locus.pl?locus=YOR128C)*-101* [*can1*](http://db.yeastgenome.org/cgi-bin/locus.pl?locus=YEL063C) [*his3*](http://db.yeastgenome.org/cgi-bin/locus.pl?locus=YOR202W) [*gal2*](http://db.yeastgenome.org/cgi-bin/locus.pl?locus=YLR081W) [*trp1*](http://db.yeastgenome.org/cgi-bin/locus.pl?locus=YDR007W)*-289* [*tyr1*](http://db.yeastgenome.org/cgi-bin/locus.pl?locus=YBR166C) [*ura3*](http://db.yeastgenome.org/cgi-bin/locus.pl?locus=YEL021W)*-52* [*pol2*](http://db.yeastgenome.org/cgi-bin/locus.pl?locus=YNL262W)*-11* | [4] |
| *pol2-16* | 3 | *MAT*α *trp1-1 ura3-1 his3-11,15 leu2-3,112 ade2-1 can1-100 bar1::*  *hisG pol2-16* | [4] |
| *pol2-18* | 2 | *MAT*α *ade5 leu2 trp1 ura3 pol2-3::LEU2 [YCp-pol2-18 (TRP1)]* | [4] |

1. Osborn AJ, Elledge SJ: **Mrc1 is a replication fork component whose phosphorylation in response to DNA replication stress activates Rad53**. *Genes Dev* 2003, **17**:1755–1767.

2. Giaever G, Chu AM, Ni L, Connelly C, Riles L, Véronneau S, Dow S, Lucau-Danila A, Anderson K, André B, Arkin AP, Astromoff A, El-Bakkoury M, Bangham R, Benito R, Brachat S, Campanaro S, Curtiss M, Davis K, Deutschbauer A, Entian K-D, Flaherty P, Foury F, Garfinkel DJ, Gerstein M, Gotte D, Güldener U, Hegemann JH, Hempel S, Herman Z, et al.: **Functional profiling of the Saccharomyces cerevisiae genome.** *Nature* 2002, **418**:387–91.

3. Naylor ML, Li JM, Osborn AJ, Elledge SJ: **Mrc1 phosphorylation in response to DNA replication stress is required for Mec1 accumulation at the stalled fork**. *Proc Natl Acad Sci U S A* 2009, **106**:12765–12770.

4. Lou H, Komata M, Katou Y, Guan Z, Reis CC, Budd M, Shirahige K, Campbell JL: **Mrc1 and DNA polymerase epsilon function together in linking DNA replication and the S phase checkpoint**. *Mol Cell* 2008, **32**:106–117.
